# Two new species of genus *Oecleopsis* Emeljanov from China, with descriptions of female genitalia of five species (Hemiptera, Fulgoromorpha, Cixiidae)

**DOI:** 10.3897/zookeys.768.24796

**Published:** 2018-06-19

**Authors:** Yan Zhi, Lin Yang, Pei Zhang, Xiang-Sheng Chen

**Affiliations:** 1 Institute of Entomology, Guizhou University, Guiyang, Guizhou, 550025, P.R. China; 2 The Provincial Special Key Laboratory for Development and Utilization of Insect Resources of Guizhou, Guizhou University, Guiyang, Guizhou, 550025, P.R. China; 3 Laboratory Animal Center, Guizhou Medical University, Guiyang, Guizhou, 550025, P.R. China; 4 Xingyi Normal University for Nationalities, Xingyi, Guizhou, 562400, P.R. China

**Keywords:** Female genitalia, Fulgoroidea, morphology, Pentastirini, planthopper, taxonomy

## Abstract

Two new species of genus *Oecleopsis* Emeljanov, 1971, *O. laminatus* Zhi & Chen, **sp. n.** and *O. productus* Zhi & Chen, **sp. n.**, and a new record, *O. yoshikawai* (Ishihara, 1961), are described and illustrated from China. Female genitalia of five species within this genus are compared morphologically: *O. laminatus* Zhi & Chen, **sp. n.**, *O. mori* (Matsumura, 1914), *O. productus* Zhi & Chen, **sp. n.**, *O. sinicus* (Jacobi, 1944) and *O. yoshikawai* (Ishihara, 1961). A key to five Chinese species based on female genitalia, and a key to all known species of *Oecleopsis* based on male genitalia, are provided.

## Introduction

Emeljanov (1971) established the cixiid planthopper genus *Oecleopsis* with *Oliarus artemisiae* Matsumura, 1914 as its type species. This genus belongs to the tribe Pentastirini of subfamily Cixiinae (Hemiptera: Cixiidae). Van Stalle (1991) and Guo et al. (2009) studied this genus, bringing the total of known species to twelve: *Oecleopsis artemisiae* (Matsumura, 1914), *O. articara* Van Stalle, 1991, *O. bifidus* (Tsaur, Hsu & Van Stalle, 1988), *O. chiangi* (Tsaur, Hsu & Van Stalle, 1988), *O. elevatus* (Tsaur, Hsu & Van Stalle, 1988), *O. mori* (Matsumura, 1914), *O. petasatus* (Noualhier, 1896), *O. sinicus* (Jacobi, 1944), *O. spinosus* Guo, Wang & Feng, 2009, *O. tiantaiensis* Guo, Wang & Feng, 2009, *O. wuyiensis* Guo, Wang & Feng, 2009 and *O. yoshikawai* (Ishihara, 1961).

Herein, two new species: *Oecleopsis laminatus* Zhi & Chen, sp. n. and *O. productus* Zhi & Chen, sp. n. are described and illustrated from China (Yunnan province), and *O. yoshikawai* (Ishihara, 1961) is recorded from China for the first time. The genus now contains fourteen species, and all species from China.

Female genitalia of *Oecleopsis* are known relatively little: genitalia in ventral view and anal segment in dorsal view of *O. articara*, *O. sinicus*, *O. spinosus* and *O. tiantaiensis* and the anal segment of *O. mori* were illustrated; *O. bifidus*, O. el*evatus*, *O. petasatus* and *O. wuyiensis* were described briefly (Fennah 1956; Tsaur et al. 1988; Van Stalle 1991; Guo et al. 2009). These characters of external genitalia are not eﬀective to distinguish among species of *Oecleopsis*. Zhi et al. (2017) discussed external and internal structures of female genitalia in cixiid planthoppers and found that the characteristics of posterior vaginal walls can be considered as key diagnostic features for female identification in genus *Neocarpia*. Using the characters of posterior vagina in species identification is also practicable in genus *Oecleopsis* by comparing female genital morphological features of five species (other species are not included, as we do not have the female specimens): *Oecleopsis laminatus*, *O. mori*, *O. productus*, *O. sinicus* and *O. yoshikawai*. A key to five Chinese species based on female genitalia, and a key to all known species of *Oecleopsis* based on male genitalia, are provided.

## Materials and methods

The morphological terminology and measurements follow Tsaur et al. (1988), Bourgoin et al. (2015) and Van Stalle (1991). The morphological terminology of female genitalia follows Bourgoin (1993). Body length was measured from apex of vertex to tip of forewing; vertex length was measured the median length of vertex (from apical transverse carina to tip of basal emargination). External morphology and drawings were done with the aid of a Leica MZ 12.5 stereomicroscope. Photographs of female genitalia were taken with Nikon SMZ25 and other photographs with KEYENCE VHX-1000 system. Illustrations were scanned with CanoScan LiDE 200 and imported into Adobe Photoshop CS7 for labeling and plate composition. The dissected male genitalia are preserved in glycerine in small plastic tubes pinned together with the specimens.

The type specimens examined are deposited in the Institute of Entomology, Guizhou University, Guiyang, Guizhou Province, China (**GUGC**).

## Taxonomy

### Oecleopsis

Taxon classificationAnimaliaHemipteraCixiidae

Emeljanov, 1971

Oecleopsis Emeljanov, 1971: 621; Anufriev and Emeljanov 1988: 460; Van Stalle 1991: 20; Guo et al. 2009: 46.

#### Type species.

*Oliarus artemisiae* Matsumura, 1914, original designation.

For the relationship and diagnosis of *Oecleopsis* see Van Stalle (1991: 20) and Guo et al. (2009: 46).

#### Distributions.

China, Japan, Korea, Russia, Thailand, Malaya, Cambodia, Borneo.

#### Checklist and distributions of species of *Oecleopsis*

*O. artemisiae* (Matsumura, 1914); China (Sichuan), Japan, Korea, Russia (Kunashir Island).

*O. articara* Van Stalle, 1991; China (Hainan, Henan, Sichuan), Malaya, Borneo.

*O. bifidus* (Tsaur, Hsu & Van Stalle, 1988); China (Fujian, Taiwan).

*O. chiangi* (Tsaur, Hsu & Van Stalle, 1988); China (Taiwan).

*O. elevatus* (Tsaur, Hsu & Van Stalle, 1988); China (Guizhou, Taiwan), Japan (Honshu).

*O. laminatus* Zhi & Chen, sp. n.; China (Yunnan).

*O. mori* (Matsumura, 1914); China (Guangxi, Taiwan, Yunnan).

*O. productus* Zhi & Chen, sp. n.; China (Yunnan).

*O. petasatus* (Noualhier, 1896); China (Hainan, Sichuan, Yunnan), Cambodia.

*O. sinicus* (Jacobi, 1944); China (Anhui, Fujian, Guangxi, Guizhou, Henan, Hunan, Shanxi, Sichuan), Japan.

*O. spinosus* Guo, Wang & Feng, 2009; China (Shaanxi).

*O. tiantaiensis* Guo, Wang & Feng, 2009; China (Shaanxi).

*O. wuyiensis* Guo, Wang & Feng, 2009; China (Fujian, Henan, Hunan, Shaanxi).

*O. yoshikawai* (Ishihara, 1961); China (Guizhou), Thailand (Chiengmai).

#### Key to five Chinese species (females) of *Oecleopsis*

**Table:** 

1	Basal sclerite on right side of posterior vagina with a process ventrally	**2**
–	Basal sclerite on right side of posterior vagina without process ventrally (Figs 63–64)	***O. sinicus***
2	Right side of posterior vagina with a triangular sclerite ventrally (Figs 51–52)	***O. laminatus* sp. n.**
–	Right side of posterior vagina without triangular sclerite ventrally	**3**
3	Terminal area on right side of posterior vagina with a long and narrow sclerite, curved, towards the right ventrally (Figs 55–56)	***O. mori***
	Without the same sclerite ventrally	**4**
4	Right side of posterior vagina with one sclerite, which constricted medially in ventral view (Figs 59–60)	***O. productus* sp. n.**
–	Right side of posterior vagina with two sclerites, terminal irregular sclerite forming three projecting oblong structures in ventral view (Figs 67–68)	***O. yoshikawai***

#### Key to species (males) of the genus *Oecleopsis* (revised from Van Stalle (1991) and Guo et al. (2009))

**Table:** 

1	Spinose process on right side near apex of periandrium long, longer than 1/2 length of periandrium	**2**
–	Spinose process on right side near apex of periandrium short, not longer than 1/4 length of periandrium	**9**
2	Apex of flagellum circular	**3**
–	Apex of flagellum not circular	**4**
3	Left side near apex of periandrium with a short spinose process; dorsal margin of flagellum with a long spinose process and left side with a short process (Figs 31–34)	***O. productus* sp. n.**
–	Left side of periandrium without spinose process; dorsal margin of flagellum without process and left side with a long process (Van Stalle 1991: fig. 79)	***O. articara***
4	Apical process of flagellum not bifurcated (Figs 45–48)	***O. yoshikawai***
–	Apical process of flagellum bifurcated	**5**
5	Flagellum with a large laminal process (Figs 17–20)	***O. laminatus* sp. n.**
–	Flagellum without laminal process	**6**
6	Left side near base of flagellum with a spinose process (Van Stalle 1991: fig. 72)	***O. petasatus***
–	Left side of flagellum without process	**7**
7	Flagellum with one subapical process (Tsaur et al. 1988: fig. 9 (C–D))	***O. bifidus***
–	Flagellum with two subapical processes	**8**
8	Two subapical processes of flagellum short, much shorter than apical process (Anufriev and Emeljanov 1988: fig. 358; Van Stalle 1991: fig. 101)	***O. artemisiae***
–	Two subapical processes of flagellum long, nearly equal to apical process in length (Tsaur et al. 1988: fig. 10 (F–G))	***O. elevatus***
9	Apical process of flagellum not bifurcated	**10**
–	Apical process of flagellum bifurcated	**11**
10	Spinose process on right side of periandrium rather short, directed ventrally; vertex 2.6 times longer than wide (Guo et al. 2009: fig. 10–11)	***O. spinosus***
–	Spinose process on right side of periandrium longer, directed dorsocephalically; vertex 1.5 times longer than wide (Tsaur et al. 1988: fig. 8 (C–D))	***O. chiangi***
11	Rami of bifurcation symmetrical, almost equal in length (Van Stalle 1991: fig. 92)	***O. sinicus***
–	Rami of bifurcation asymmetrical, unequal in length	**12**
12	Left ramus of bifurcation rudimentary, only a small protuberance (Guo et al. 2009: figs 20–21)	***O. tiantaiensis***
–	Left ramus of bifurcation well developed	**13**
13	Length of right ramus of apical process about 1.8 times as long as that of left ramus, ventral margin near base of periandrium with a spinose process (Guo et al. 2009: figs 31–32)	***O. wuyiensis***
–	Length of right ramus of apical process about 3.0 times as long as that of left ramus, ventral margin near base of periandrium without process (Van Stalle 1991: fig. 85)	***O. mori***

### Oecleopsis laminatus

Taxon classificationAnimaliaHemipteraCixiidae

Zhi & Chen sp. n.

http://zoobank.org/D59DDB8B-7041-46DD-A5A5-E197C5B049FA

Figs 1–2 , 7–20 , 49–52

#### Type material.

Holotype: ♂, **China**: Yunnan Province, Jingdong County, Taizhong Town (24°30'N, 100°56'E), 18 August 2009, Bin Zhang; paratypes: 1♂, 2♀♀, same data as holotype; 1♀, Yunnan Province, Jingdong County, Dajie Town, 22 August 2009, Bin Zhang; 3♂♂, 2♀♀, Yunnan Province, Lanping County, Lajin Town, 4 August 2012, Yong-Gang Xiao.

#### Description.

Body length: male 6.7–7.7mm (*N* = 5), female 7.0–9.0mm (*N* = 5).

**Figures 1–6.**
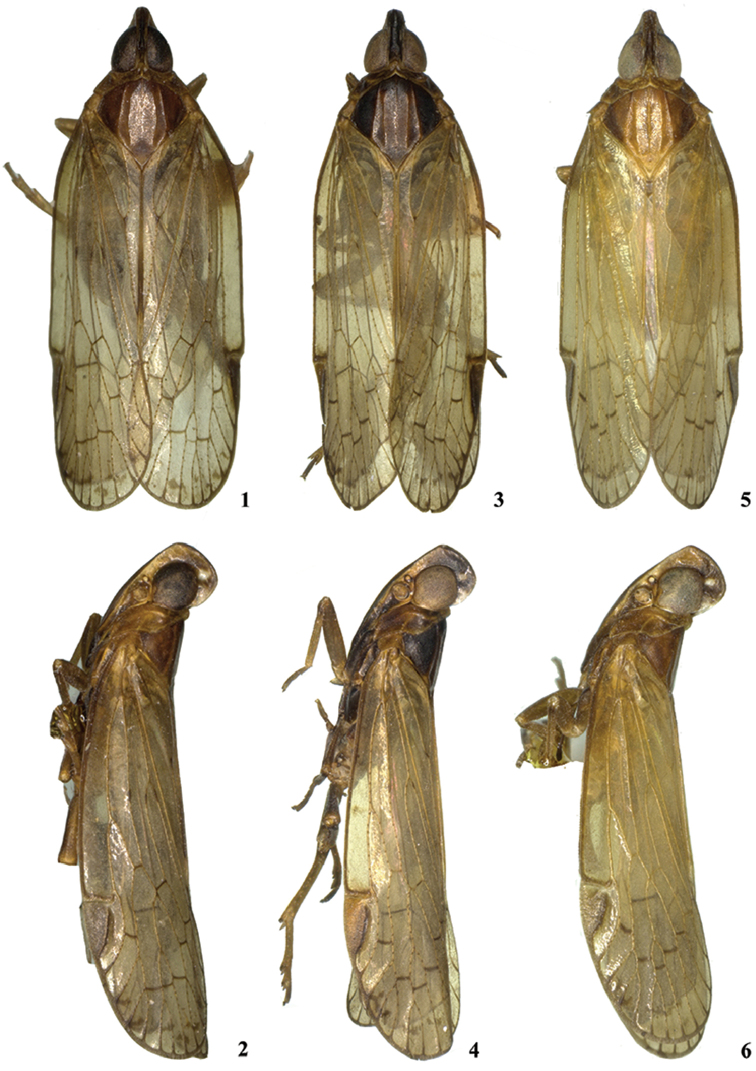
*Oecleopsis* species (male). **1–2** *O. laminatus* sp. n. **1** dorsal view **2** lateral view **3–4** *O. productus* sp. n. **3** dorsal view **4** lateral view **5–6** *O. yoshikawai* **5** dorsal view **6** lateral view.

**Figures 7–20.**
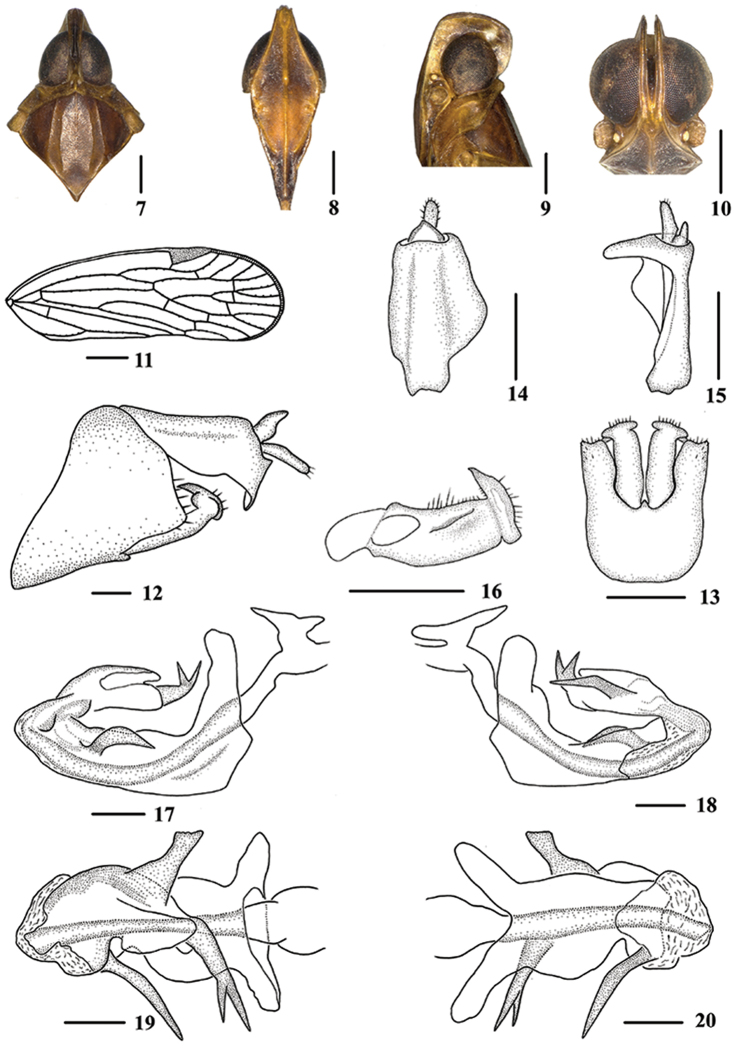
*Oecleopsis laminatus* sp. n., male **7** Head and thorax, dorsal view **8** Face, ventral view **9–10** Head **11** Forewing **12** Genitalia, lateral view **13** Pygofer and genital styles, ventral view **14–15** Anal segment **14** dorsal view **15** right lateral view **16** Genital styles, inner lateral view **17** Aedeagus, right side **18** Aedeagus, left side **19** Aedeagus, dorsal view **20** Aedeagus, ventral view. Scale bars: 0.5 mm (**7–10, 12–20**); 1.0 mm (**11**).

*Coloration.* General color mid brown (Figs 1–2, 7–10). Eyes mid brown, ocelli yellowish brown. Vertex blackish brown. Face yellowish to blackish brown, carinae lighter; rostrum blackish brown. Pronotum mid to blackish brown with carinae yellowish or light brown; mesonotum mid to blackish brown, sometimes area between lateral carinae lighter. Forewing semi-translucent, light brown, stigma yellowish or mid brown. Hind tibiae and abdominal sternites mid brown.

*Head and thorax*. Vertex (Fig. 7) narrow, 4.3 times longer than wide; Frons (Fig. 8) 1.3 times as long as wide. Forewing (Fig. 11) 3.0 times longer than wide, with 11 apical and 6 subapical cells; fork Sc+RP distal to fork CuA1+CuA2; RP 3 branches, MA 3 branches, MP 2 branches. Hind tibia with 3–4 lateral spines; chaetotaxy of hind tarsi: 7/5.

*Male genitalia*. Pygofer (Figs 12–13) dorsal margin concave and U-shaped ventrally, widened towards apex; in lateral view, lateral lobes triangularly extended caudally. Medioventral process triangular ventrally. Anal segment (Figs 12, 14–15) tubular, asymmetrical, widened towards apex in left side view; in right side view, left ventral margin convex and right ventral margin excavated near apex; 1.9 times longer than wide in dorsal view; anal style fingerlike, beyond anal segment. Genital styles illustrated in Fig. 16. Aedeagus (Figs 17–20) in total with four processes. Spinose process on right side near apex of periandrium long, directed right side, more than 1/2 length of periandrium. Flagellum terminating into a bifurcate process, asymmetrical; left side near apex of flagellum with a large laminal process, apex transversal and directed left side.

*Female genitalia*. Genitalia as shown in Fig. 49 ventrally. Anal tube (Fig. 50) 1.8 times longer than wide in dorsal view. Posterior vagina (Figs 51–52) elongate, with a long longitudinal sclerite dorsally, large. In ventral view, left side with a long longitudinal sclerite; right side with a rhombic sclerite basally, which with a process, the process directed left-ventrally and longer than shown in the figure, terminal area with a triangular sclerite.

#### Distribution.

China (Yunnan).

#### Etymology.

The specific name is derived from the Latin word “*laminatus*”, referring to the left side of flagellum with a large laminal process.

#### Remarks.

Male genitalia of *O. laminatus* sp. n. is similar to *O. bifidus*, but differs in: (1) left side near apex of flagellum with a laminal process, apex transversal and directed left side (in *O. bifidus*, with a spinose process, apex pointed, curved right-dorsally); (2) process on right side of periandrium directed right side (directed left side slightly in *O. bifidus*); (3) left ramus of bifurcate process longer than right one (in *O. bifidus*, left ramus of bifurcate process shorter than right one); (4) vertex rather narrow, about 4.3 times longer than wide (in *O. bifidus*, about 1.6 times).

Female genitalia of *O. laminatus* sp. n. is similar to *O. mori*, but differs in: (1) terminal area on right side of posterior vagina with a triangular sclerite in *O. laminatus* (the sclerite long and narrow, curved, towards the right in *O. mori*); (2) left side of posterior vagina with a straight sclerite ventrally in *O. laminatus* (the sclerite in the same position much longer and curved, towards the right in *O. mori*).

### Oecleopsis mori

Taxon classificationAnimaliaHemipteraCixiidae

(Matsumura, 1914)

Figs 53–56

Oliarus mori Matsumura, 1914: 426; Tsaur et al. 1988: 48, fig. 7 (A–G).
Oecleopsis mori (Matsumura, 1914): Van Stalle 1991: 23, figs 85–91; Guo et al. 2009: 50.

#### Material examined.

**China**: 7♂♂, 11♀♀, Guangxi Province, Jinxiu County, Lianhua Mountain, 30 April 2011, Xiao-Fei Yu, Rong Huang; 3♂♂, 2♀♀, Guangxi Province, Wuming County, Daming Mountain, 14 May 2011, Rong Huang; 6♂♂, 12♀♀, Guangxi Province, Wuming County, Daming Mountain, 14–15 May 2012, Rong Huang, Hu Li, Wei-Cheng Yang, Zhi-Hua Fan.

#### Supplementary description.

*Female genitalia*. Genitalia as shown in Fig. 53 ventrally. Anal tube (Fig. 54) 1.8 times longer than wide in dorsal view. Posterior vagina (Figs 55–56) elongate, with a long longitudinal sclerite dorsally, large. In ventral view, left side with a long longitudinal sclerite, curved, towards the right; right side with a longitudinal sclerite basally, which with a torsional oblong sclerite and a process, the process directed left-ventrally and longer than shown in the figure; terminal area with a long and narrow sclerite, curved, towards the right.

#### Distributions.

China (Guangxi, Taiwan, Yunnan).

#### Note.

The female genitalia of this species is described and illustrated in detail for the first time.

### Oecleopsis productus

Taxon classificationAnimaliaHemipteraCixiidae

Zhi & Chen sp. n.

http://zoobank.org/AD015429-772E-4B7F-809D-5CD8CBE35EF9

Figs 3–4 , 21–34 , 57–60

#### Type material.

Holotype: ♂, **China**: Yunnan Province, Baoshan, Baihua ridge (25°17'N, 98°48'E), 15 June 2011, Jian-Kun Long; paratypes: 1♂, 14♀♀, same data as holotype, 13–15 June 2011; 3♂♂, 1♀, collection area same as holotype, 6 May 2010, Bin Zhang, Yan-Li Zheng; 2♂♂, 1♀, Fugong County, Yunnan Province, 17 May 2011, Bin Zhang, Yan-Li Zheng, Bin Yan.

#### Description.

Body length: male 6.7–6.9mm (*N* = 7), female 6.7–7.8mm (*N* = 16).

**Figures 21–34.**
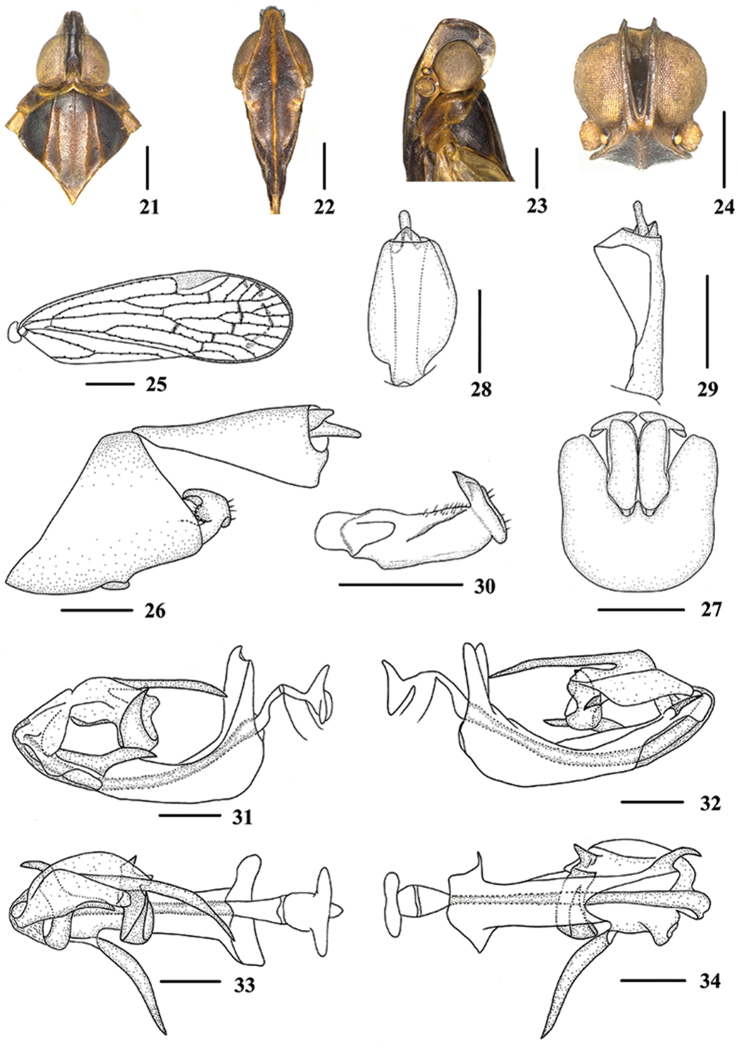
*Oecleopsis productus* sp. n., male **21** Head and thorax, dorsal view **22** Face, ventral view **23–24** Head **25** Forewing **26** Genitalia, lateral view **27** Pygofer and genital styles, ventral view **28–29** Anal segment **28** dorsal view **29** right lateral view **30** Genital styles, inner lateral view **31** Aedeagus, right side **32** Aedeagus, left side **33** Aedeagus, dorsal view **34** Aedeagus, ventral view. Scale bars: 0.5 mm (**21–24, 26–34**); 1.0 mm (**25**).

*Coloration.* General color blackish brown (Figs 3–4, 21–24). Eyes mid brown, ocelli light yellow. Vertex blackish brown. Face mid to blackish brown, carinae lighter; rostrum mid to blackish brown. Pronotum mid to blackish brown with carinae lighter; mesonotum mid to blackish brown, area between lateral carinae lighter. Forewing semi-translucent, light brown, stigma mid brown. Hind tibiae and abdominal sternites blackish brown.

*Head and thorax*. Vertex (Fig. 21) narrow, 3.5 times longer than wide; frons (Fig. 22) 1.7 times as long as wide. Forewing (Fig. 25) 3.0 times longer than wide, with 12 apical and 6 subapical cells; fork Sc+RP distal to fork CuA1+CuA2; RP 4 branches, MA 3 branches, MP 2 branches. Hind tibia with 4 lateral spines; chaetotaxy of hind tarsi: 7/5.

*Male genitalia*. Pygofer (Figs 26–27), dorsal margin concave and U-shaped ventrally, widened towards apex; in lateral view, lateral lobes triangularly extended caudally. Medioventral process narrowly triangular ventrally. Anal segment (Figs 26, 28–29) tubular, asymmetrical, widened towards apex in left side view; in right side view, left ventral margin convex and right ventral margin excavated near apex; 1.8 times longer than wide in dorsal view; anal style fingerlike, beyond anal segment. Genital styles illustrated in Fig. 30. Aedeagus (Figs 31–34) in total with five processes. Spinose process on right side near apex of periandrium long, directed right-dorsocephalically, more than 1/2 of periandrium in length; left side near apex of periandrium with a short reversed spinose process, curving slightly, directed dorsocaudally. Flagellum tapering, apex curved in a semi-circle; a long spinose process arising from dorsal margin, reaching to basal margin of periandrium, curving right side, directed ventrocephalically; left side with a short triangular process, directed ventrocephalically.

*Female genitalia*. Genitalia as shown in Fig. 57 ventrally. Anal tube (Fig. 58) 1.9 times longer than wide in dorsal view. Posterior vagina (Figs 59–60) elongate, with a long longitudinal sclerite dorsally, large. In ventral view, left side with a long longitudinal sclerite, semi-sclerotized, right side with a large median constricted sclerite, which with a process, the process directed left-ventrally and longer than shown in the figure.

#### Distribution.

China (Yunnan).

#### Etymology.

The specific name is derived from the Latin *productus*, referring to the dorsal margin of flagellum with a long process.

#### Remarks.

Male genitalia of *O. productus* sp. n. is similar to *O. articara*, but differs in: (1) left side near apex of periandrium with a short process (in *O. articara*, left side without process); (2) a very long spinose process arising from dorsal margin of flagellum (dorsal margin of flagellum without process in *O. articara*); (3) left side near apex of flagellum with a very short process (in *O. articara*, left side near middle of flagellum with a much longer process); 4) forewing with RP 4 branches (in *O. articara*, 3 branches).

Female genitalia of *O. productus* sp. n. is similar to *O. yoshikawai*, but differs in: (1) right side of posterior vagina with one sclerite, which constricted medially in ventral view in *O. productus* (with two sclerites, terminal irregular sclerite forming three projecting oblong structures in ventral view in *O. yoshikawai*); (2) in ventral view, distal part of the sclerite on left side of posterior vagina not extended and curved to right in *O. productus* (extended and curved to right in *O. yoshikawai*).

### Oecleopsis sinicus

Taxon classificationAnimaliaHemipteraCixiidae

(Jacobi, 1944)

Figs 61–64 , 69

Mnemosyne sinica Jacobi, 1944: 12.
Oliarus sinicus (Jacobi, 1944): Van Stalle 1988: 46.
Oliarus cucullatus Noualhier, 1896: 255; Fennah 1956: 453, fig. 3(G–H) (misidentification); Chou et al. 1985: 23, fig. 20 (misidentification).
Oecleopsis sinicus (Jacobi, 1944), Van Stalle 1991: 23, figs 92–100; Guo et al. 2009, 51, figs 1–6.

#### Material examined.

**China**: 3♂♂, 2♀♀, Shanxi Province, Lishan National Nature Reserve (1300–2200m), 12–15 July 2012, Xiao-Hui Hou; 2♂♂, Anhui province, Huangshan city, Tangkou town (500m), 20 May 2008, Zheng-Guang Zhang; 9♂♂, 4♀♀, Fujian province, Shanghang county, Natural Reserve of Meihua Mountain, Gutian town, 17 August 2009, Pei Zhang, Jun-Qiang Ni; 3♂♂, 5♀♀, Henan province, Jiyuan City, Wangwu Mountain, 22 August 2009, Yu-Jian Li; 2♂♂, Sichuan province, Yaan city, Zhougong Mountain, 15–18 July 2010, Pei Zhang; 1♂, Sichuan Province, Tianquan County, Labahe, 25 July 2012, Zhi Hua Fan; 21♂♂, 15♀♀, Sichuan Province, Mianyang City, Anzhou District, Chaping, 19–22 July 2010, Pei Zhang, Yan-Li Zheng, Ke-Bin Li, Zhi-Min Chang; 5♂♂, 2♀♀, Sichuan Province, Mianyang City, Wanglang National Nature Reserve, Changbaigou (2587m), 25 July 2016, Meng-Shu Dong; 17♂♂, 6♀♀, Guizhou province, Wangmo county, Fuxing Town, 9 August 2012, Wei-Chen Yang; 7♂♂, 13♀♀, Guizhou province, Wangmo county, Dayi Town, 15–16 July 2016, Liang-Jing Yang, Yong-Shun Ding; 14♂♂, 3♀♀, Guizhou province, Wangmo county, Dayi Town, 25–26 June 2013, Jian-Kun Long, Ji-Chun Xing, Hai-Yan Sun; 11♂♂, 4♀♀, Guizhou province, Maolan county, Banzhai Town, 5–6 July 2010, Xiao-Hui Hou, Pei Zhang; 4♂♂, 1♀, Guizhou province, Bijie City, Bazhai Town, Jinyin mountain, 7 July 1977, collector unknown; 11♂♂, 14♀♀, Guizhou Province, Weining County, Caohai National Natural Reserve, 1–6 August 2017, Ying-Jian Wang, Liang-Jing Yang, Nian Gong, Guan-Fu Ma; 1♂, Guizhou province, Rongjiang county, Pingyang Town, Xiaodanjiang, 9 July 2011, Jian-Kun Long; 1♂, 1♀, Guizhou province, Luodian county, Moyang Town, 10 May 2013, Zhi-Hua Fan.

#### Supplementary description.

*Female genitalia*. Genitalia as shown in Fig. 61 ventrally. Anal tube (Fig. 62) 2.1 times longer than wide in dorsal view. Posterior vagina (Figs 63–64) elongate, with a longitudinal sclerite respectively dorsally and medially. In ventral view, right side with a more or less triangular sclerite basally, terminal area with a transverse long sclerite.

#### Host plant.

*Artemisia* sp. (Compositae); *Zea mays* Linnaeus (Panicoideae).

#### Distributions.

China (Anhui, Fujian, Guangxi, Guizhou, Henan, Hunan, Shanxi, Sichuan), Japan.

#### Remarks.

Female genitalia of *Oecleopsis sinicus* can be distinguished from other species of the genus by the following characters: basal sclerite on right side of posterior vagina without process ventrally; terminal area with a transverse long sclerite ventrally.

#### Note.

The female genitalia of this species is described and illustrated in detail for the first time.

### Oecleopsis yoshikawai

Taxon classificationAnimaliaHemipteraCixiidae

(Ishihara, 1961)

Figs 5–6 , 35–48 , 65–68

Oliarus yoshikawai Ishihara, 1961: 228, figs 6–7.
Oecleopsis yoshikawai (Ishihara, 1961): Van Stalle 1991: 22, figs 65–71; Guo et al. 2009, 58.

#### Material examined.

**China**: 2♂♂, 2♀♀, Guizhou province, Wangmo county, Xintun Town, Bakang Village, 19 August 2012, Jian-Kun Long, Shi-Yan Xu, Wei-Bin Zheng; 2♂♂, 2♀♀, Guizhou province, Wangmo county, Xintun Town, Bakang Village, 2013-VI-28, Jian-Kun Long, Hai-Yan Sun, Yang-Yang Liu; 1♂, Guizhou province, Anlong county, Xianheping Nature Reserve, 22 July 2016, Liang-Jing Yang; 1♀, Guizhou province, Wangmo county, Zhexiang Town, 19 July 2016, Liang-Jing Yang; 1♂, Guizhou province, Guanling county, Bangui Town, 20 August 2009, Ji-Chun Xing.

#### Redescription.

Body length: male 6.0–6.5mm (*N* = 6), female 6.8–7.5mm (*N* = 5).

**Figures 35–48.**
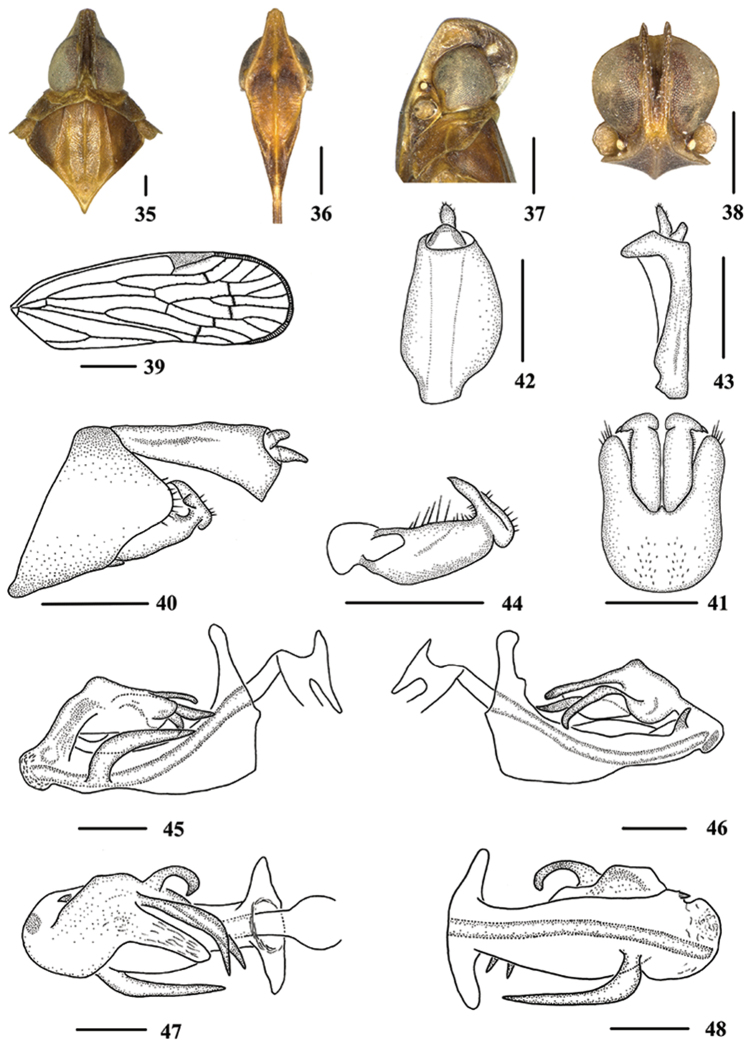
*Oecleopsis yoshikawai* (Ishihara, 1961), male **35** Head and thorax, dorsal view **36** Face, ventral view **37–38** Head **39** Forewing **40** Genitalia, lateral view **41** Pygofer and genital styles, ventral view **42–43** Anal segment **42** dorsal view **43** right lateral view **44** Genital styles, inner lateral view **45** Aedeagus, right side **46** Aedeagus, left side **47** Aedeagus, dorsal view **48** Aedeagus, ventral view. Scale bars: 0.5 mm (**35–38, 40–48**); 1.0 mm (**39**).

*Coloration.* General color mid brown (Figs 5–6, 35–38). Eyes mid to blackish brown, ocelli light yellow. Vertex blackish brown. Face yellowish to blackish brown, carinae yellowish to mid brown; rostrum yellowish to blackish brown. Pronotum mid to blackish brown; mesonotum yellowish to mid brown. Forewing semitranslucent, light brown, stigma mid brown. Hind tibiae and abdominal sternites mid brown.

*Head and thorax*. Vertex (Fig. 35) narrow, 3.6 times longer than wide; frons (Fig. 36) 1.2 times as long as wide. Forewing (Fig. 39) 3.2 times longer than wide, with 11 apical and 6 subapical cells; fork Sc+RP slightly distal to fork CuA1+CuA2; RP 3 branches, MA 3 branches, MP 2 branches. Hind tibia with 3 lateral spines; chaetotaxy of hind tarsi: 7/5.

*Male genitalia*. Pygofer (Figs 40–41), dorsal margin concave and U-shaped ventrally, widened towards apex; in lateral view, lateral lobes triangularly extended caudally, apex round. Medioventral process triangular ventrally, short. Anal segment (Figs 40, 42–43) tubular, asymmetrical, widened towards apex in left side view; in right side view, left ventral margin convex and right ventral margin excavated near apex; 2.0 times longer than wide in dorsal view; anal style fingerlike, beyond anal segment. Genital styles illustrated in Fig. 44. Aedeagus (Figs 45–48) in total with five processes. Spinose process on right side near apex of periandrium long, basal part curving upward and distal part parallel to periandrium, directed towards cephalum, more than 1/2 of periandrium in length; left side near apex of periandrium with a short spinose process, curved and directed dorsocaudally. Flagellum bearing three spinose processes: the upper one originating the middle of flagellum, apex curved strongly and directed right side; the apical one curved right-ventrocephalically; the one on left side shortest, curved strongly and directed ventrally.

*Female genitalia*. Genitalia as shown in Fig. 65 ventrally. Anal tube (Fig. 66) 1.8 times longer than wide in dorsal view. Posterior vagina (Figs 67–68) elongate, with a long longitudinal sclerite dorsally. In ventral view, left side with a long longitudinal sclerite, curved, towards the right; right side with a longitudinal sclerite basally, which with a process, the process directed left-ventrally and longer than shown in the figure; terminal area with an irregular sclerite, which forming three projecting oblong structures.

**Figures 49–68.**
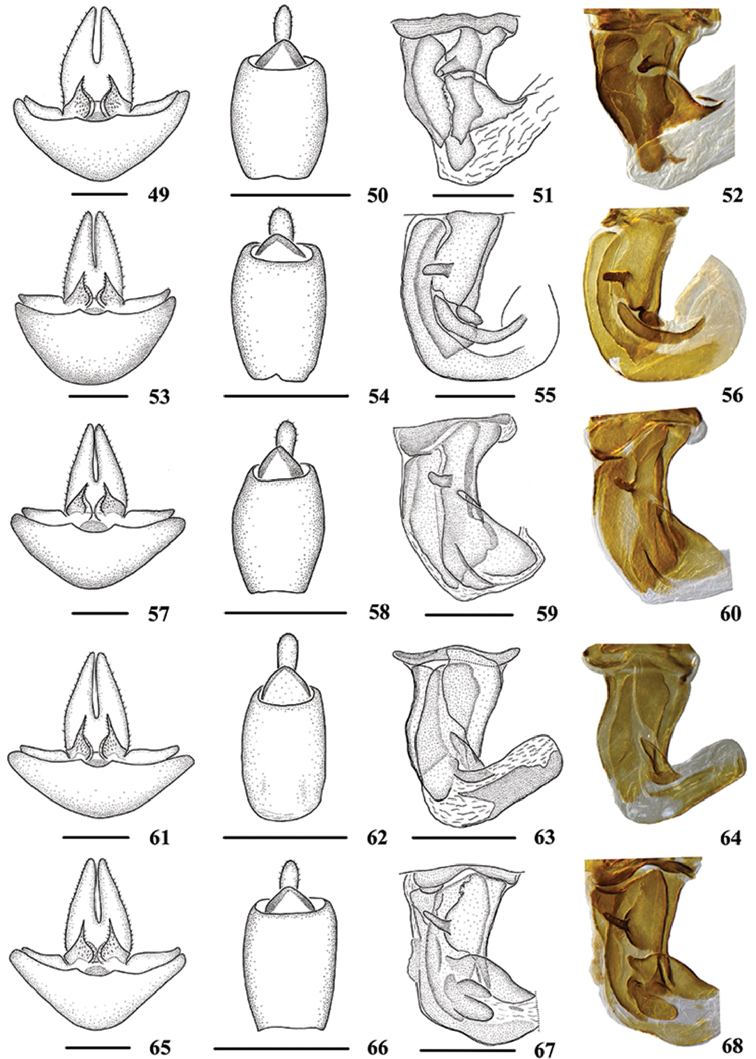
Female genitalia of *Oecleopsis* species. **49–52** *O. laminatus* sp. n. **53–56** *O. mori* **57–60** *O. productus* sp. n. **61–64** *O. sinicus* **65–68** *O. yoshikawai* **49, 53, 57, 61, 65** Genitalia, ventral view **50, 54, 58, 62, 66** Anal segment, dorsal view **51–52, 55–56, 59–60, 63–64, 67–68** Posterior vagina, ventral view. Scale bars: 0.5 mm.

**Figure 69.**
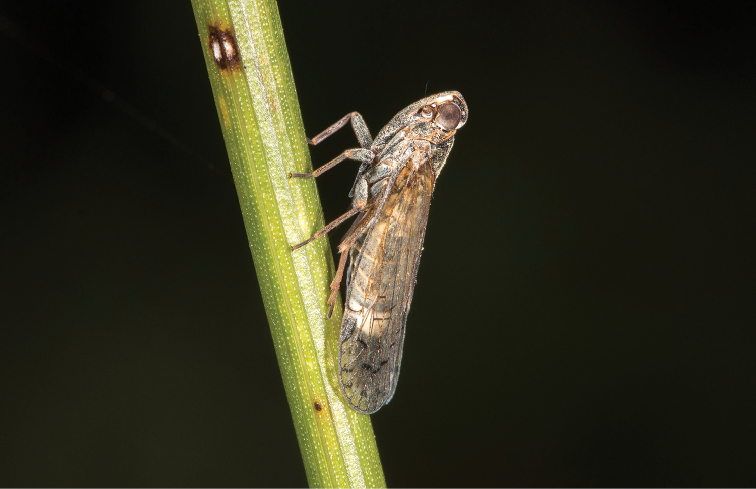
Adult of *Oecleopsis sinicus* (Jacobi, 1944), lateral view, female (Caohai National Natural Reserve, Weining County, Guizhou Province, 1 August 2017, photograph by X-S Chen).

#### Host plant.

Bamboo (Bambusoideae).

#### Distributions.

China (Guizhou), Thailand.

#### Note.

This species is recorded from China for the first time and the female genitalia of this species is described and illustrated for the first time.

## Supplementary Material

XML Treatment for Oecleopsis

XML Treatment for Oecleopsis laminatus

XML Treatment for Oecleopsis mori

XML Treatment for Oecleopsis productus

XML Treatment for Oecleopsis sinicus

XML Treatment for Oecleopsis yoshikawai
